# Functional characterization of novel *MFSD8* pathogenic variants anticipates neurological involvement in juvenile isolated maculopathy

**DOI:** 10.1111/cge.13673

**Published:** 2019-12-12

**Authors:** Miriam Bauwens, Stephan Storch, Nicole Weisschuh, Chantal Ceuterick‐de Groote, Riet De Rycke, Brecht Guillemyn, Sarah De Jaegere, Frauke Coppieters, Rudy Van Coster, Bart P. Leroy, Elfride De Baere

**Affiliations:** ^1^ Center for Medical Genetics Ghent University Ghent Belgium; ^2^ Department of Biochemistry, Children's Hospital University Medical Center Hamburg‐Eppendorf Hamburg Germany; ^3^ Molecular Genetics Laboratory, Institute for Ophthalmic Research University of Tuebingen Tuebingen Germany; ^4^ Laboratory of Neuromuscular Pathology Institute Born‐Bunge (IBB) and University of Antwerp Antwerp Belgium; ^5^ Department of Biomedical Molecular Biology Ghent University Ghent Belgium; ^6^ VIB‐UGent Center for Inflammation Research Ghent Belgium; ^7^ Ghent University Expertise Centre for Transmission Electron Microscopy and VIB BioImaging Core Ghent Belgium; ^8^ Center for Medical Genetics Ghent University Hospital Ghent Belgium; ^9^ Department of Pediatrics, Division of Pediatric Neurology and Metabolism Ghent University Hospital Ghent Belgium; ^10^ Department of Ophthalmology Ghent University and Ghent University Hospital Ghent Belgium; ^11^ Division of Ophthalmology and Center for Cellular & Molecular Therapeutics The Children's Hospital of Philadelphia Philadelphia Pennsylvania

**Keywords:** CLN7, functional studies, inherited retinal disease, locus resequencing of *ABCA4*, maculopathy, *MFSD8* variants, neuronal ceroid lipofuscinosis, whole exome sequencing

## Abstract

Biallelic *MFSD8* variants are an established cause of severe late‐infantile subtype of neuronal ceroid lipofuscinosis (v‐LINCL), a severe lysosomal storage disorder, but have also been associated with nonsyndromic adult‐onset maculopathy. Here, we functionally characterized two novel *MFSD8* variants found in a child with juvenile isolated maculopathy, in order to establish a refined prognosis. *ABCA4* locus resequencing was followed by the analysis of other inherited retinal disease genes by whole exome sequencing (WES). Minigene assays and cDNA sequencing were used to assess the effect of a novel *MFSD8* splice variant. *MFSD8* expression was quantified with qPCR and overexpression studies were analyzed by immunoblotting. Transmission electron microscopy (TEM) was performed on a skin biopsy and ophthalmological and neurological re‐examinations were conducted. WES revealed two novel *MFSD8* variants: c.[590del];[439+3A>C] p.[Gly197Valfs*2];[Ile67Glufs*3]. Characterization of the c.439+3A>C variant via splice assays showed exon‐skipping (p.Ile67Glufs*3), while overexpression studies of the corresponding protein indicated expression of a truncated polypeptide. In addition, a significantly reduced *MFSD8* RNA expression was noted in patient's lymphocytes. TEM of a skin biopsy revealed typical v‐LINCL lipopigment inclusions while neurological imaging of the proband displayed subtle cerebellar atrophy. Functional characterization demonstrated the pathogenicity of two novel *MFSD8* variants, found in a child with an initial diagnosis of juvenile isolated maculopathy but likely evolving to v‐LINCL with a protracted disease course. Our study allowed a refined neurological prognosis in the proband and expands the natural history of *MFSD8*‐associated disease.

## Graphical abstract

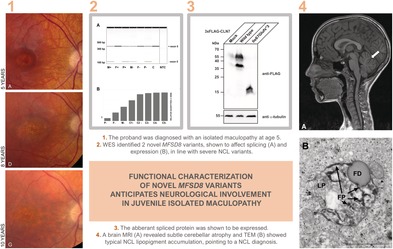

## 1 INTRODUCTION

Isolated macular dystrophies are characterized by degeneration of the central inner retina. Up to now, isolated maculopathies were found to be associated with over 26 genes and 2 loci, of which ATP binding cassette subfamily A member 4 gene (*ABCA4*) is the most frequently involved and the Major Facilitator Superfamily Domain containing Protein‐8 gene (*MFSD8*, encoding CLN7) one of the more recently identified genes.^1^, ^2^, ^3^, ^4^ The associated phenotype of the latter is characterized by macular dystrophy with central cone involvement or widespread retinopathy. This has been attributed to the combination of hypomorphic and loss‐of‐function *MFSD8* alleles or biallelic mild *MFSD8* alleles.^1^, ^2^, ^3^ Biallelic loss‐of‐function *MFSD8* variants on other hand, display a subtype of neuronal ceroid lipofuscinosis (NCL), named variant late‐infantile NCL (v‐LINCL, CLN7, NCL7), which is a severe lysosomal storage disorder leading to neurodegeneration.^5^, ^6^, ^7^ The first NCL symptoms usually arise between 2 and 5 years of age and are characterized by epileptic seizures and developmental regression.^8^ Ultimately ataxia, myoclonus, and visual impairment are seen, which are typical features of a progressive NCL disease leading to premature death. As in other NCL subtypes, accumulation of autofluorescent storage material in neurons and in other cell types can sometimes be observed, ranging from fingerprint and curvilinear structures to rectilinear profiles.^9^, ^10^

Here, the female proband presented with an isolated maculopathy initially diagnosed as atypical Stargardt disease at age 5 and underwent genetic testing of the entire *ABCA4* gene, followed by whole exome sequencing (WES)‐based inherited retinal disease gene panel testing. Identification of novel *MFSD8* variants and their downstream functional characterization led to ophthalmological and neurological reassessments, finally allowing refinement of the neurological prognosis of this proband and expanding the natural history of *MFSD8*‐associated disease.

## 2 MATERIALS AND METHODS

### 2.1 Subjects

Legal parental consent was obtained for the study, which was approved by the institutional ethical committee (B670201525349 and B670201734438). The proband is a currently 12‐year‐old Caucasian female, with nonconsanguineous parents. She was first noted to have visual problems at age 5, and was originally diagnosed with atypical Stargardt disease.

### 2.2 Clinical assessment of the proband

Ophthalmic examination of the female proband at age 5 included best‐corrected visual acuity (BCVA) measurements, fundus photography, and electroretinography (ERG) according to the International Society for Clinical Electrophysiology of Vision (ISCEV) standards. In addition, spectral‐domain optical coherence tomography (SD‐OCT) and blue light (488 nm) autofluorescence imaging (BAF), Heidelberg Spectralis HRA+OCT (Heidelberg Engineering, Heidelberg, Germany) were performed. Ophthalmologic reassessments were undertaken at ages 8 and 10. In addition, a neurological assessment was performed, consisting of a clinical neurological examination, electroencephalogram (EEG) and brain magnetic resonance imaging (MRI).

### 2.3 Molecular genetic analyses

EDTA blood samples were obtained from the proband, the unaffected parents and five healthy controls. DNA was isolated from peripheral blood lymphocytes by standard procedures. In addition, short‐term lymphocyte cultures were established for all samples.

#### 2.3.1 Targeted next‐generation sequencing of the coding region of ABCA4

The coding region of *ABCA4* was enriched by PCR amplification of all coding exons and flanking splice‐site sequences, followed by targeted next‐generation sequencing (NGS) as described (MiSeq, Illumina, San Diego, California).^11^

#### 2.3.2 Locus resequencing of ABCA4

A region encompassing the entire *ABCA4* gene (chr1:94337885‐94703604, hg19) was enriched using a custom HaloPlex Target enrichment kit (Agilent Technologies, Belgium), followed by NGS (MiSeq, Illumina, San Diego, California). Data were analyzed as described previously.^12^

#### 2.3.3 Whole exome sequencing

To enrich and sequence the exome, the SureSelectXT HumanAllExon V5+UTRs kit (Agilent, Santa Clara, California) and NextSeq 500 (Illumina, San Diego, California) were used. Data were mapped with the CLC Bio software (CLC Bio, Qiagen, Hilden, Germany) and analyzed using the Ingenuity Variant Analysis pipeline (Qiagen, Hilden, Germany). Keywords used for filtering were Stargardt disease, blindness, and macular degeneration. Sanger sequencing was used to confirm and assess segregation of the filtered variants, in both the proband and the parents (Tables S1 and S2).

#### 2.3.4 RPGR ORF15 testing

Whole exome sequencing‐based testing was complemented with *RPGR* ORF15 testing. Targeted enrichment of ORF15 amplicons using PCR was followed by library preparation (Nextera XT, Illumina, San Diego, California) and NGS (Miseq, Illumina, San Diego, California).

### 2.4 Transcript analyses

#### 2.4.1 cDNA sequencing

Short‐term lymphocyte cultures were treated either with or without puromycin as a nonsense‐mediated decay inhibitor (200 μg/ml; Sigma‐Aldrich, St. Louis, Missouri) 5 hours before RNA extraction. RNA isolation was followed by DNase treatment (Heat & Run gDNA removal kit, ArticZymes, Tromsø, Norway) and cDNA synthesis (iScript cDNA synthesis kit, BioRad, Hercules, California). After PCR, RT‐PCR products were loaded onto the capillary labchip (Caliper, Hopkinton, Massachusetts) and Sanger sequenced (ABI3730XL, Genetic analyser; Applied Biosystems, Foster City, California). The PCR primers can be found in Table S3.

#### 2.4.2 Expression analysis

Quantitative polymerase chain reaction (qPCR; LC480, Roche, Basel, Switzerland) was used to compare RNA expression levels of *MFSD8* between the proband, the carrier parents and five healthy controls. Primers can be found in Table S4. qBasePlus (Biogazelle, Zwijnaarde, Belgium) was used for data‐analysis, normalization was performed using two reference genes (*YWAZ* and *GAPDH*).

### 2.5 In vitro splice assays

A genomic segment of *MFSD8* spanning exon 5 (hg19, chr4:128864292‐128865864) was amplified from patient DNA using primers with *Not*I and *Bam*HI recognition sequences (Table S5). PCR products were cloned into the pCR2.1 plasmid (Invitrogen‐Life Technologies, Carlsbad, California). Wild type (WT) and mutant inserts were excised by digestion and subcloned into digested pSPL3_2096 (Invitrogen‐Life Technologies, Carlsbad, California). HEK293T cells were transfected with pSPL3‐*MFSD8* constructs using Lipofectamine (Invitrogen‐Life Technologies, Carlsbad, California). Total RNA was extracted after 24 hours (peqGOLD Total RNA Kit, VWR, Radnor, Pennsylvania) and reversely transcribed (Transcriptor High Fidelity cDNA Synthesis Kit, Roche Applied Science, Penzberg, Germany). The cDNA was PCR amplified with pSPL3 exon primers and Sanger sequenced (Applied Biosystems, Foster City, California).

### 2.6 In vitro overexpression studies

#### 2.6.1 Cloning of 3xFLAG CLN7 expression constructs

A cDNA construct with a triple FLAG tag fused to the N‐terminus of CLN7 was generated by amplifying human *MFSD8* cDNA (NM_152778, RZPD clone IRATp970E0532D6; imaGenes, Berlin, Germany) using primers 3xFLAG pCMV10‐CLN7 F/R and cloning this into the p3xFLAG‐CMV10 vector (Sigma‐Aldrich, St. Louis, Missouri). The cDNA coding for human mutant CLN7 Ile67Glufs*3 was purchased (Source BioScience, Nottingham, UK) and amplified. Primers can be found in Table S6. PCR products were separated and purified from agarose gels, cleaved with restriction enzymes and cloned into the p3xFLAG‐CMV10 expression vector (Sigma‐Aldrich, St. Louis, Missouri) generating the mutant 3xFLAG p.Ile67Glufs*3.

#### 2.6.2 Western blot analysis

HEK293T cells were transiently transfected using Jet Pei reagent (Polyplus Transfection, Illkrich, France) with either 3xFLAG‐CLN7, 3xFLAG‐p.Ile67Glufs*3 or a mock construct as control and harvested 24 hours after the start of transfection. Preparation of postnuclear supernatants and total membrane fractions was performed as described previously.^13^ Membrane pellets were homogenized in extraction buffer containing 50 mM Tris‐HCl, pH 7.5, 1% TX‐100 (v/v), 1 mM EDTA and inhibitor cocktail and incubated on ice for 30 minutes. Immunoblot analyses were performed using anti‐FLAG and anti‐α‐tubulin antibodies (Sigma, St. Louis, Missouri).

### 2.7 Transmission electron microscopy

A skin biopsy from the patient was explanted and incubated at 37°C in a humidified atmosphere containing 5% CO_2_. Part of this biopsy was fixed and processed for transmission electron microscopy (TEM), using standard techniques: fixation in 2.5% glutaraldehyde and 4% formaldehyde, postfixation in osmium tetroxide, embedding in araldite, staining with uranyl acetate and lead citrate. An FEI CM10 transmission electron microscope was used at 60 kV.

## 3 RESULTS

### 3.1 Identification of biallelic novel *MFSD8* variants

Given the initial diagnosis of atypical Stargardt disease in the female proband at age 5, targeted testing of *ABCA4* was conducted. Sequencing of the coding region of *ABCA4* and of the entire *ABCA4* locus revealed one heterozygous *ABCA4* variant c.3113C>T; p.(Ala1038Val). No other (likely) pathogenic coding or noncoding *ABCA4* variants including copy number variants were identified. Subsequently, WES was performed in the proband and data were filtered in order to select variants in known retinopathy genes, assuming an autosomal recessive inheritance mode (Table S2). WES was complemented with screening of ORF15 of the *RPGR* gene, which was negative. Two novel heterozygous *MFSD8* variants were identified in the proband: c.590del and c.439+3A>C. Both unaffected parents were shown to be heterozygous carrier of an *MFSD8* variant. They were not observed in the NCL database containing all published NCL variants (http://www.ucl.ac.uk/ncl/cln7.shtml) nor in dbSNP (http://www.ncbi.nlm.nih.gov/SNP/), 1000Genomes (http://www.1000genomes.org/), gnomAD (http://gnomad.broadinstitute.org/), or GoNL (http://www.nlgenome.nl/). Only the c.590del variant was found in the ExAC browser (ALL: 0.00082%, SAS: 0.0061%). The c.590del variant, located in exon 7, has the same deleterious prediction on protein level (p.(Gly197Valfs*2)) as the known pathogenic *MFSD8* c.588del variant.

### 3.2 Characterization of the novel *MFSD8* variant c.439+3A>C

#### 3.2.1 Splicing assessment

**Figure 1:**
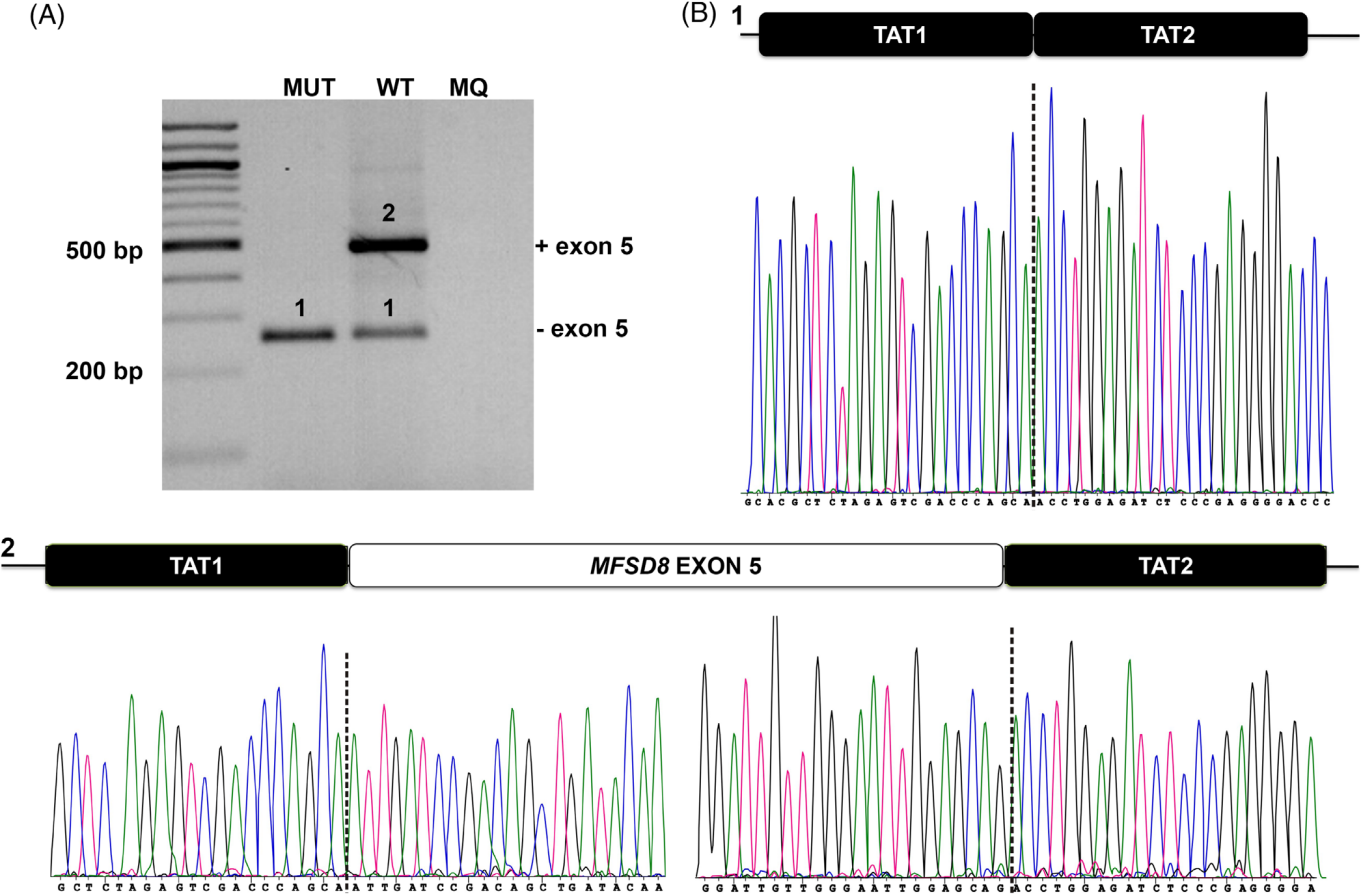
The *MFSD8* variant c.439+3A>C decreases the strength of the canonical donor site of exon 5, leading to a skip of exon 5, here shown by a minigene assay. The minigene vector pSPL3, containing two HIV‐TAT exons (TAT1, TAT2) flanking the insert and containing the RT‐PCR primer sequences was used. A, RT‐PCR products were loaded on gel. MUT (1): RT‐PCR product produced by mutant construct; c.439+3C, WT (1 and 2): RT‐PCR products produced by wild type construct; c.439+3A, MQ: milliQ, RT‐PCR product of a no‐template control RT‐PCR reaction. B, Sanger sequencing profiles of the different RT‐PCR products (1 and 2) confirms skipping of exon 5

**Figure 2:**
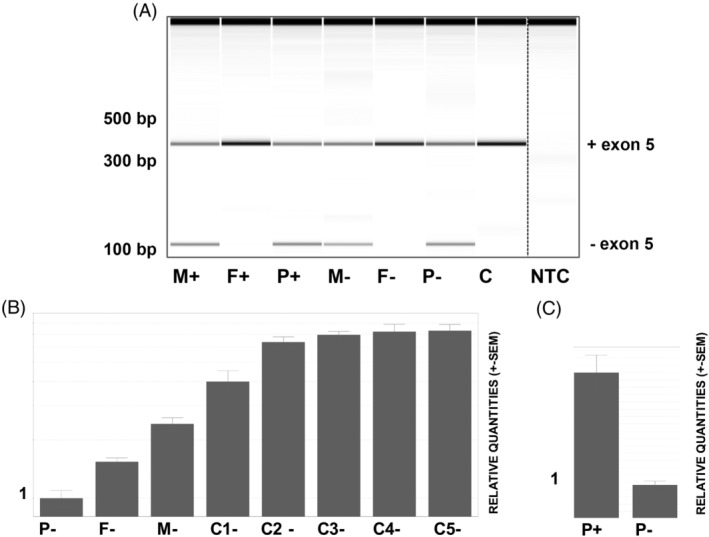
A, Lymphocyte cDNA derived from both patient (P) and parents (M: mother and F: father) confirms the exon‐skip in the patient and carrier mother. This was seen with (+) and without puromycin (−) supplementation. The normal cDNA PCR product, containing exon 5, is 341 bp in size, while the exon 5 loss created a product that is 241 bp smaller. B, The normalized expression profiles of patient (P) and both parents (F and M) are severely reduced when compared to controls (C1‐C5). C, *MFSD8* mRNA expression levels in the patient are higher with puromycin (+) addition. C, negative control, NTC, no‐template control; SEM, standard error of the mean

The different splice annotations of the c.439+3A>C variant pointed toward a subtle weakening of the donor site of exon 5. A minigene assay revealed that this variant leads to a skip of exon 5 (241 bp) at the RNA level in all resulting transcripts, as opposed to the WT situation, where exon 5 skipping is seen in a smaller fraction of the transcript (Figure 1). This out‐of‐frame exon‐skip was confirmed on lymphocyte cDNA from the proband and the carrier mother while no exon‐skipping could be observed in the noncarrier father or control (Figure 2A). Contrary to the minigene results, no endogenous exon 5 skipping was observed in noncarriers. Next, the combined and individual effects of both variants on *MFSD8* expression was assessed using qPCR on lymphocyte cDNA of the proband, the carrier parents and five controls. A strong reduction of *MFSD8* mRNA expression was seen in the proband and her carrier parents as compared to the controls, with the strongest reduction observed in the proband with biallelic *MFSD8* variants (Figure 2B‐C).

#### 3.2.2 Overexpression studies

**Figure 3:**
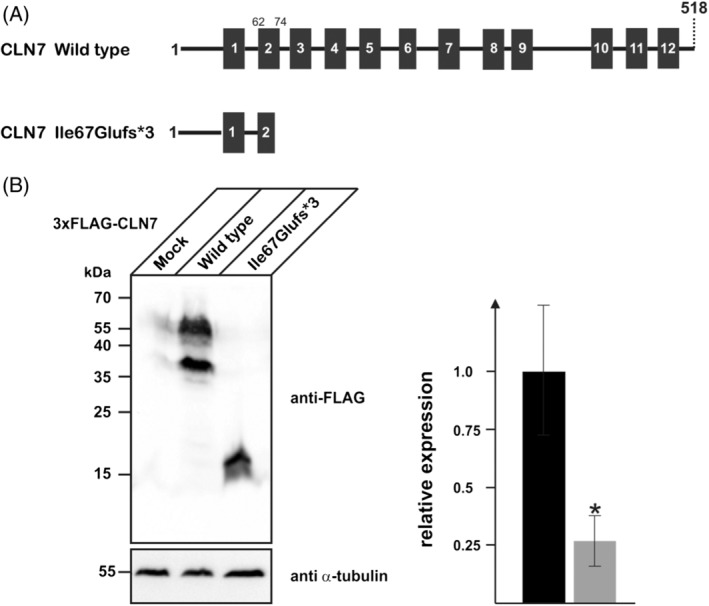
A, The novel *MFSD8* variant c.439+3A>C leads to an exon 5 skip, resulting in a truncated protein (p.Ile67Glufs*3). A full length CLN7 protein contains 12 transmembrane domains, the truncated protein p.Ile67Glufs*3 stops within transmembrane domain 2. B, Western blot results of overexpression studies (performed in triplicate). Membrane fractions (120 μg protein) were analyzed by FLAG immunoblotting. Αlpha‐tubulin Western blot was performed to control equal loading. Positions of the molecular mass markers are indicated. In extracts from 3xFLAG CLN7 expressing HEK293T cells, the full length CLN7 as well as the cleaved N‐terminal fragment could be detected, here visible as two bands. In 3xFLAG p.Ile67Glufs*3 expressing cells, a truncated 15 kDa CLN7 protein could be detected. The bar diagram represents relative expression levels of wild type (black bar) and mutant p.Ile67Glufs*3 (gray bar) normalized to the levels of α‐tubulin from three independent transfections (*p ˂ .05)

In order to study the effect of the c.439+3A>C variant at the protein level, overexpression studies with the truncated CLN7 protein fused to an N‐terminal triple FLAG tag (3xFLAG p.Ile67Glufs*3) were performed in HEK293T cells, followed by FLAG immunoblotting of membrane fractions (Figure 3). In 3xFLAG CLN7 expressing HEK293T cells, the full length CLN7 as well as the cleaved N‐terminal fragment were detected, in contrast to 3xFLAG p.Ile67Glufs*3 expressing cells where a truncated 15 kDa CLN7 protein was detected.^14^ These data suggest that the truncated CLN7 polypeptide is expressed, albeit at lower levels compared to WT CLN7 protein and that it is detectable in membrane fractions. Based on previously published results of truncated CLN7 protein, it can be predicted that Cln7p.Ile67Glufs*3 is integrated into membranes but most likely is retained in the endoplasmic reticulum.^14^

### 3.3 Ultrastructural features

**Figure 4:**
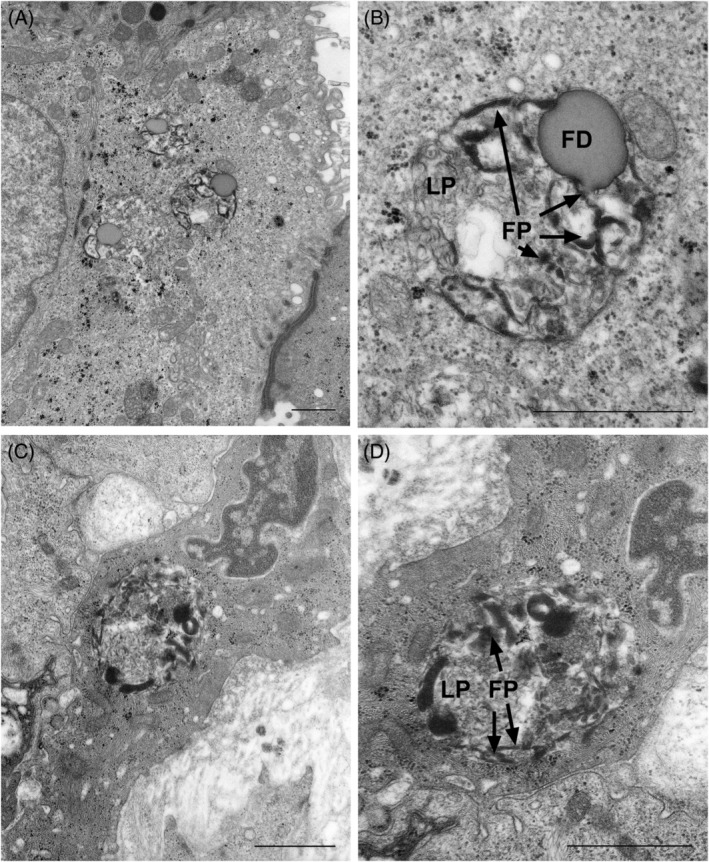
Transmission electron microscopy images taken from a skin biopsy of the proband (scalebar: 1 μm). A, Inclusions can be seen in an eccrine sweat gland (enlargement: 14 500×). B, Detail of image A: membrane‐enclosed inclusion with a heterogeneous aspect; lamellar profiles (LP), fingerprints (FP, arrows) and a fat droplet (FD) are visible. C, Pleomorphic inclusion in an endothelial cell (blood vessel; enlargement: 27 000×). D, Detail of C: inclusion with fingerprints (FP, arrows) and mixed lamellar profiles (LP; enlargement: 41 000×)

Transmission electron microscopy revealed characteristic membrane‐bounded inclusions in different cell types of the skin. In secretory cells of the eccrine sweat glands and blood vessels (endothelial and smooth muscle cells) many pleomorphic inclusions (1‐2 μm diameter), could be particularly noted, showing mixed rectilinear, curvilinear, and fingerprint lamellar profiles (Figure 4). Some of the inclusions displayed a more pseudovacuolar aspect (2‐3 μm diameter).

### 3.4 Clinical neurological and ophthalmological assessments

#### 3.4.1 Ophthalmological assessment

**Figure 5:**
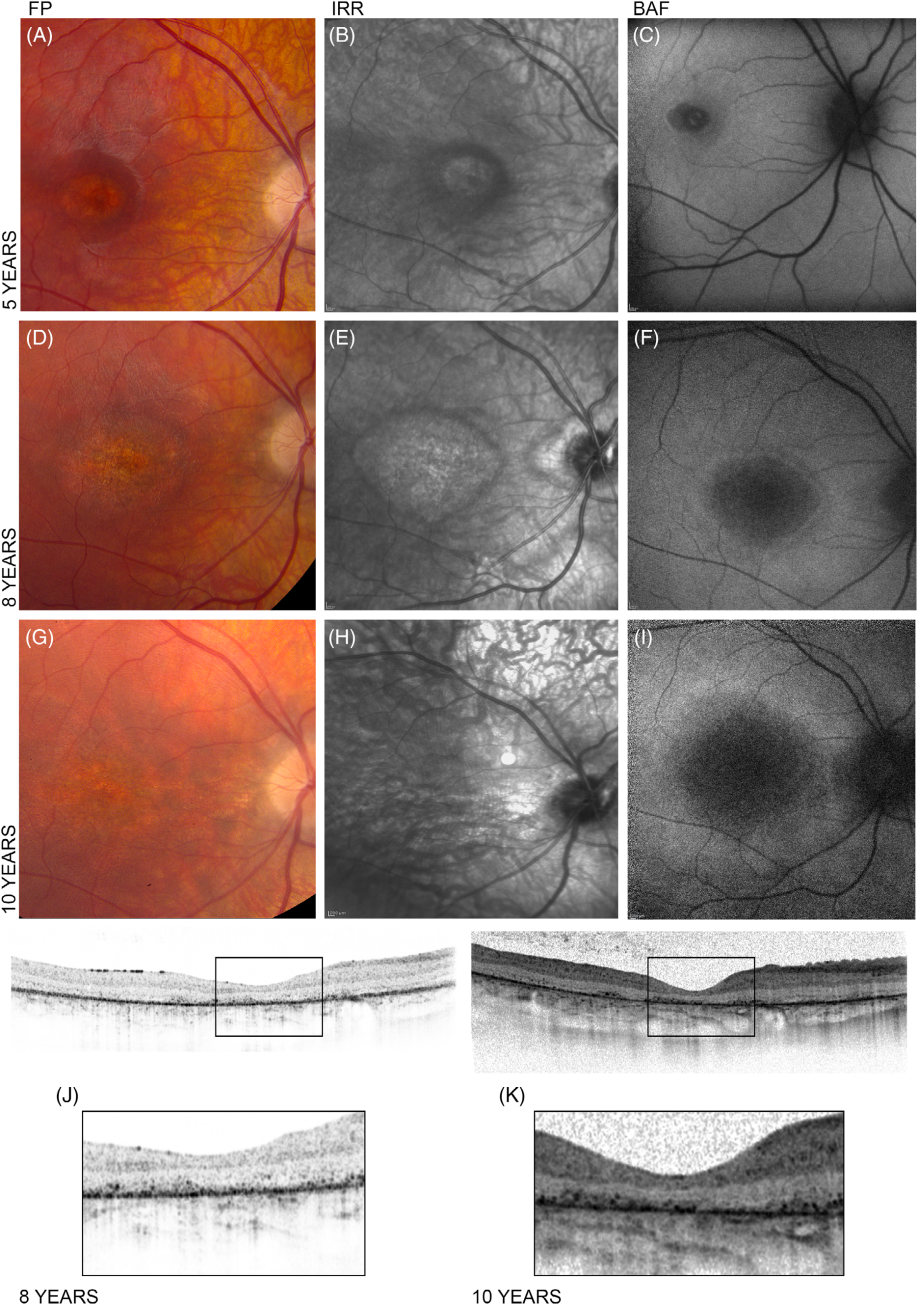
A‐C, Ophthalmological assessment at age 5 showed central macular abnormalities, no fishtail flecks were observed. D‐F, Reassessment at age 8 revealed expansion of the central macular atrophic area (right eye). G‐I, This expansion was even more widespread at age 10 (right eye). J and K, Optical coherence tomography images taken at age 8 and 10 clearly show the evolution of the atrophy of the outer retinal area. BAF, blue light; FP, fundus photography; IRR, near‐infrared radiation

The patient was initially referred with a diagnosis of Stargardt disease at the age of five. Six months prior to this first visit a subnormal BCVA was noted. BCVA was measured at a Snellen equivalent at 0.13 in both eyes, without correction. Fundus examination showed central macular abnormalities in keeping with outer retinal atrophy. No fishtail flecks typical of Stargardt disease were observed (Figure 5A‐C). OCT of the macula at age 5 years showed selective thinning of the outer retinal layers, more pronounced of those representing the photoreceptors. This area of atrophy was generally broader than what is typical seen at an early stage Stargardt.

**Figure 6:**
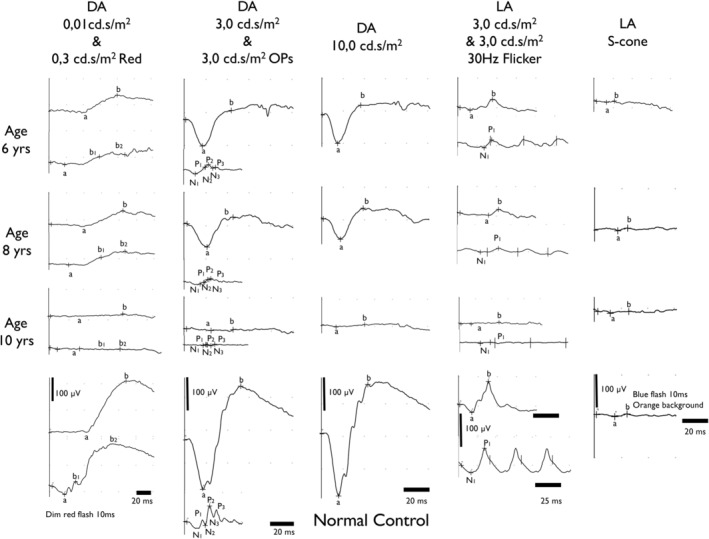
A full‐field flash ERG at ages 6 and 8 was in keeping with a generalized rod‐cone dystrophy with additional post‐photoreceptor involvement as evidenced by a more pronounced reduction of the b‐wave than the a‐wave in the maximal combined rod‐cone response (reduced b/a‐ratio). At age 10, a rapid decline of generalized retinal function is illustrated by complete absence of all retinal responses on full‐field flash ERG. DA, dark‐adapted; LA, light‐adapted. ERG, electroretinography

Three years later, at age 8, BCVA had decreased to 0.07 in both eyes. Funduscopy showed expansion of the central macular atrophic area surrounded by a hyperautofluorescent ring on blue light autofluorescence imaging (Figure 5D‐F). A full‐field flash electroretinogram (ERG) at both ages was in keeping with a generalized rod‐cone dystrophy with additional post‐photoreceptor involvement as evidenced by a more pronounced reduction of the b‐wave than the a‐wave in the maximal combined rod‐cone response (reduced b/a‐ratio; Figure 6). Evolution was very limited between ages 6 and 8. An OCT at age 8 showed that the area of outer retinal atrophy in the macula had increased, in keeping with the increase of the funduscopic lesion (Figure 5J).

At age 10, vision was decreased to counting fingers in both eyes, white light and infrared reflectance fundus photography show further expansion of the area of macular atrophy (Figure 5G‐I). Rapid decline of generalized retinal function was illustrated by complete absence of all retinal responses on full‐field flash ERG (Figure 6). Also, further extension of the area of outer retinal atrophy in the macula was visible on OCT (Figure 5K). Extensive retinopathy is reflected by a less well‐defined hyperautofluorescent ring in central macula, surrounded by more diffuse hyperautofluorescence within and beyond vascular arcades, including the retinal midperiphery. Near‐infrared reflectance imaging shows fine vertical striations in the interpapillomacular bundle, as previously described in mid to later stage *CLN3*‐related maculopathy by Dulz et al.^15^ These striations are visible on OCT as fine cobblestone‐like abnormalities of the inner limiting membrane.

#### 3.4.2 Neurological assessment

**Figure 7:**
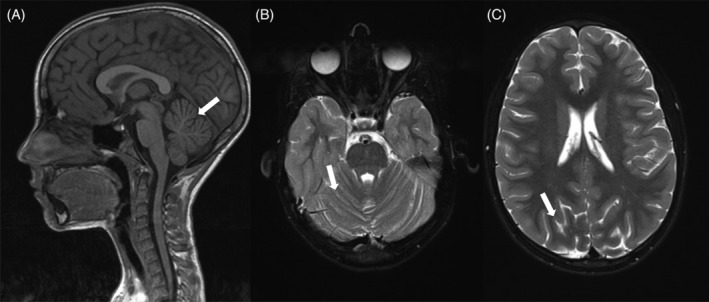
A‐B, MRI images, taken at age 8. Cerebellar atrophy is visible on T1 and T2 weighted images (arrow). C, In addition, subtle atrophy of the supratentorial cortex can been seen (arrow). MRI, magnetic resonance imaging

After the identification of the two *MFSD8* variants a neurological investigation was undertaken. An EEG did not show any clear epileptiformic discharges, even after intermittent light stimulation. At first glance, the MRI results were classified as normal. However, upon further inspection of T2 weighted images, signs of cerebellar atrophy were visible, as well as mild atrophy of the supratentorial cortex. The cerebellar atrophy was also visible on T1 weighted sagittal images (Figure 7A‐C). Currently, at age 12, no epileptic seizures or ataxia have occurred.

## 4 DISCUSSION

Using WES, we identified two novel *MFSD8* variants c.590del p.(Gly197Valfs*2) and c.439+3A>C p.(Ile67Glufs*3) in a now 12‐year‐old proband with isolated maculopathy, initially diagnosed as atypical Stargardt disease. Publications by Roosing et al, Khan et al, and Birtel et al revealed that the combination of a loss‐of‐function allele with a hypomorphic allele or homozygosity for a mild allele in *MFSD8* might give rise to an isolated ocular phenotype ranging from localized maculopathy to widespread retinopathy with severe macular involvement or retinitis pigmentosa (RP).^1^, ^2^, ^3^ Interestingly, a recent publication by Zare‐Abdollahi et al reported for the first time patients with an isolated maculopathy and with known compound heterozygous and homozygous missense *MFSD8* variants, all of which have previously been solely associated with v‐LINCL.^4^

In contrast to the patient described here, in whom the first visual problems were noticed at age 5, the reported onset of the symptoms in these IRD patients occurred in late adolescence or adulthood (15‐46 years) and had a slower progression.

As the majority of the pathogenic variants in the *MFSD8* gene have been associated with the syndromic v‐LINCL phenotype, we were prompted to determine the functional impact and severity of these novel *MFSD8* variants. Based on the predictions at the protein level, the c.590del variant p.(Gly197Valfs*2), can be considered a severe mutation. Using a minigene assay and transcript analysis of lymphocyte RNA, we could establish that the other allele of interest c.439+3A>C induced skipping of exon 5 ‐although other aberrant splicing cannot be ruled out—and is predicted to result in a truncated protein p.(Ile67Glufs*3). Overexpression studies showed that the truncated protein was still expressed, but based on previously published data it is likely to be retained in the endoplasmic reticulum and does not reach the lysosomes.^14^ In addition, the proband has a reduced mRNA *MFSD8* expression profile when compared to healthy controls and her carrier parents.

The severity of these novel variants seems comparable to the v‐LINCL causing *MFSD8* variants (http://www.ucl.ac.uk/ncl/cln7.shtml). Corresponding with an alternative diagnosis of v‐LINCL, neurological tests including MRI were performed. While the clinical neurological examination of the proband was normal, brain MRI showed slight cerebellar atrophy. Currently, at age 12, no epileptic seizures or ataxia have occurred. The ultrastructural examination of a skin biopsy of the proband revealed typical mixed lipopigment inclusions in line with the observations in v‐LINCL patients.

A report illustrating atypical syndromic disease progression was published once by Kousi et al.^16^ They described a patient homozygous for p.(Ala157Pro), who first reported visual failure as the presenting symptom at 11 years of age.^16^ This was followed by motor impairment at age 24, seizures and ataxia at age 25 and 28. Several years later, mental regression and speech delay could be observed. This case eventually became chairbound by age 39.

Visual failure is usually one of the more advanced symptoms in v‐LINCL; however, several cases have been reported where visual failure precedes other symptoms such as ataxia and seizures, comparable to *CLN3* mutations that lead to Batten disease. In most of these patients, the onset of additional symptoms is delayed with 1 or 2 years when compared to other v‐LINCL patients, and seizures, mental regression and ataxia follow on average 2 years after visual symptoms are first noted.^6^, ^7^, ^16^, ^17^

At this moment, it is unclear why certain *MFSD8* missense variants act as functional null alleles and give rise to the syndromic early‐onset v‐LINCL in a similar fashion as do frameshift variants, while other missense variants, such as p.Glu336Gln, p.Met454Thr, and p.Arg482Pro are able to lead to an isolated maculopathy or retinopathy.^1^, ^2^, ^3^ A recent report on known biallelic *MFSD8* variants previously described in v‐LINCL but occurring in patients with an isolated maculopathy in this study raises even more questions and points to possible *cis*‐ or *trans*‐acting modifiers.^4^ There is no apparent link between the positions of the affected amino acids in the CLN7 protein (NP_689991.1) and the presenting symptom or disease progression, although in the latter case, these v‐LINCL‐associated variants are located in the last two exons of the gene. Localization studies have shown that none of the investigated missense variants interfere with mislocalization of the protein, but most likely are pathogenic by affecting the correct functioning of the protein, for example by aberrant proteolysis (Thr294Lys located in luminal loop 7 and Pro412Leu located in luminal loop 9).^14^

Although CLN7 is ubiquitously expressed, high expression levels have been seen in specific neuronal cell types and retinal cells, which is in line with the most severely affected tissues in v‐LINCL.^13^, ^18^, ^19^, ^20^ *MFSD8* transcripts that lack exon 2, exon 7, exons 7 and 8, and exon 11 have been described, but only the 5 kb transcript, consisting of 13 exons seems to be expressed in the brain.^5^ There is no indication that mutations in alternatively spliced exons or in surrounding introns lead to an atypical or notably milder disease course. Alternative transcripts were identified in lymphocytes and fibroblasts of patients and controls when studying the effect of splice variants near these exons (eg, c.754+2T‐A, c.863+3_4insT, and c.1102G>C).

Recently, Khan et al discussed a synaptic origin of the *MFSD8‐*associated retinal disease based on the observation that ERG abnormalities seen in the *MFSD8‐*patients reported are indicative of a post‐phototransduction abnormality and that murine MFSD8 localizes to the photoreceptor presynaptic terminals in the outer plexiform layer.^2^ The ocular phenotype in the patients studied suggests that, of all affected cell types, macular photoreceptors would be most sensitive to *MFSD8* mutations, followed by the extramacular photoreceptors, with cortical neurons being the most resistant. These observations are in line with the severity of the affected cell types and disease progression seen in the patient of this study. Our hypothesis is that in typical v‐LINCL patients, a complete lack or a minimal amount of functional CLN7 protein leads to a very rapid disease progression where there is hardly any distinction in the time of onset between the first developing symptoms and the succeeding disease features. Within some families with several affected v‐LINCL individuals having the same *MFSD8* genotype, some variability can be seen in the age of onset and sequence of symptoms. In case of residual functional CLN7 protein however, slower disease progression or even an atypical disease course is seen, with the macula being first affected and eventually leading to a more widespread retinopathy or even neuronal loss in line with the severity of the *MFSD8* pathogenic variants.

In numerous other NCL disorders, similar clinical heterogeneity was observed.^8^ A first example is CLN1 disease, were milder *CLN1* mutations, giving rise to a phenotype of later onset (late infantile, juvenile, and adult) compared to the classic infantile disease.^21^, ^22^, ^23^, ^24^ The correlation between the severity of *CLN1* mutations and onset of disease however is not fully understood, as some patients homozygous for an apparent milder variant have an earlier onset than patients who are compound heterozygous for a severe and a mild allele.^8^ There is also a spectrum of *CLN3*‐associated phenotypes, ranging from the syndromic NCL to isolated late onset RP (Wang et al) and protracted NCL with autophagic myopathy.^25^, ^26^, ^27^, ^28^, ^29^, ^30^, ^31^ Finally, *KCTD7* (CLN14) mutations were reported to underlie progressive myoclonic epilepsy and opsoclonus‐myoclonus ataxia‐like syndrome.^32^ Surprisingly, a homozygous missense variant in *KCTD7* associated with infantile onset NCL with vision loss and cognitive and motor regression has also been reported.^32^, ^33^, ^34^

In conclusion, WES‐based testing revealed two novel likely pathogenic *MFSD8* variants in a 5‐year‐old patient manifesting only visual symptoms at the time of genetic testing. Seven years later, she does not display clinically apparent neurological symptoms, which is atypical for classical v‐LINCL disease. Apart from the nonsyndromic *MFSD8*‐associated retinopathies, an atypical disease course of neurological *MFSD8*‐associated disease has been described once. Based on the young age of the patient, the functional characterization of the variants and the typical NCL storage in a skin biopsy, however, we anticipate progression to a neurodegenerative NCL‐like disorder. Finally, this study illustrates the power of integrated genomic and functional characterization for precision medicine of a heterogeneous disease, to refine clinical diagnoses and to anticipate disease progression.

## CONFLICT OF INTEREST

The authors declare no conflict of interest.

## Supporting information


**Supplementary Table S1** Primers used for *MFSD8* variant confirmation and segregation analysis.
**Supplementary Table 2**. Identified variants in IRD genes after filtering of WES results. Hez: heterozygous, AF: allele frequency (in %). *: not main transcript
**Supplementary Table 3.** Primers used for *MFSD8* cDNA sequencing.
**Supplementary Table 4**. Primers used for *MFSD8* expression analysis.
**Supplementary Table 5**. Primers used to amplify the insert cloned into a minigene construct.
**Supplementary Table 6**. Primers used to amplify cDNA for human mutant CLN7 Ile67Glufs*3

## Data Availability

The data that support the findings of this study are available on request from the corresponding author.

## References

[1] Roosing S , van den Born LI , Sangermano R , et al. Mutations in MFSD8, encoding a lysosomal membrane protein, are associated with nonsyndromic autosomal recessive macular dystrophy. Ophthalmology. 2015;122(1):170‐179. 25227500 10.1016/j.ophtha.2014.07.040

[2] Khan KN , El‐Asrag ME , Ku CA , et al. Specific alleles of CLN7/MFSD8, a protein that localizes to photoreceptor synaptic terminals, cause a spectrum of nonsyndromic retinal dystrophy. Invest Ophthalmol Vis Sci. 2017;58(7):2906‐2914. 28586915 10.1167/iovs.16-20608

[3] Birtel J , Gliem M , Mangold E , et al. Next‐generation sequencing identifies unexpected genotype‐phenotype correlations in patients with retinitis pigmentosa. PLoS One. 2018;13(12):e0207958‐e0207918. 30543658 10.1371/journal.pone.0207958 PMC6292620

[4] Zare‐Abdollahi D , Bushehri A , Alavi A , et al. MFSD8 gene mutations; evidence for phenotypic heterogeneity. Ophthal Genet. 2019;0(0):1‐5. 10.1080/13816810.2019.1592200 31006324

[5] Siintola E , Topcu M , Aula N , et al. The novel neuronal ceroid lipofuscinosis gene MFSD8 encodes a putative lysosomal transporter. Am J Hum Genet. 2007 Jul;81(1):136‐146. 17564970 10.1086/518902 PMC1950917

[6] Aiello C , Terracciano A , Simonati A , et al. Mutations in MFSD8/CLN7 are a frequent cause of variant‐late infantile neuronal ceroid lipofuscinosis. Hum Mutat. 2009;30(3):E530‐E540. 19177532 10.1002/humu.20975

[7] Aldahmesh MA , Al‐Hassnan ZN , Aldosari M , Alkuraya FS . Neuronal ceroid lipofuscinosis caused by MFSD8 mutations: a common theme emerging. Neurogenetics. 2009;10(4):307‐311. 19277732 10.1007/s10048-009-0185-1

[8] Kousi M , Lehesjoki A‐E , Mole SE . Update of the mutation spectrum and clinical correlations of over 360 mutations in eight genes that underlie the neuronal ceroid lipofuscinoses. Hum Mutat. 2011;33(1):42‐63. 21990111 10.1002/humu.21624

[9] Kohan R , Pesaola F , Guelbert N , et al. The neuronal ceroid lipofuscinoses program: a translational research experience in Argentina. BBA—molecular basis of disease. Elsevier BV. 2015;1852(PB):2301‐2311. 10.1016/j.bbadis.2015.05.003 25976102

[10] Mandel H , Katsanelson KC , Khayat M , et al. Clinico‐pathological manifestations of variant late infantile neuronal ceroid lipofuscinosis (vLINCL) caused by a novel mutation in MFSD8 gene. European. J Med Genet Elsevier Masson SAS. 2014;57(11–12):607‐612. 10.1016/j.ejmg.2014.09.004 25270050

[11] De Leeneer K , Hellemans J , Steyaert W , et al. Flexible, scalable, and efficient targeted resequencing on a benchtop sequencer for variant detection in clinical practice. Hum Mutat. 2015;36(3):379‐387. 25504618 10.1002/humu.22739

[12] Bauwens M , Garanto A , Sangermano R , et al. ABCA4‐associated disease as a model for missing heritability in autosomal recessive disorders: novel noncoding splice, cis‐regulatory, structural, and recurrent hypomorphic variants. Genet Med. 2019;21(8):1761‐1771. 30670881 10.1038/s41436-018-0420-y PMC6752479

[13] Damme M , Brandenstein L , Fehr S , et al. Gene disruption of Mfsd8 in mice provides the first animal model for CLN7 disease. Neurobiol Dis. 2014;65(C):12‐24. 24423645 10.1016/j.nbd.2014.01.003

[14] Steenhuis P , Froemming J , Reinheckel T , Storch S . Proteolytic cleavage of the disease‐related lysosomal membrane glycoprotein CLN7. BBA Mol Basis Dis. 2012;1822(10):1617‐1628. 10.1016/j.bbadis.2012.05.015 22668694

[15] Dulz S , Wagenfeld L , Nickel M , et al. Novel morphological macular findings in juvenile CLN3 disease. Br J Ophthalmol. 2016 Jun;100(6):824‐828. 26486417 10.1136/bjophthalmol-2015-307320

[16] Kousi M , Siintola E , Dvorakova L , et al. Mutations in CLN7/MFSD8 are a common cause of variant late‐infantile neuronal ceroid lipofuscinosis. Brain. 2009;132(3):810‐819. 19201763 10.1093/brain/awn366

[17] Patiño LC , Battu R , Ortega‐Recalde O , et al. Exome sequencing is an efficient tool for variant late‐infantile neuronal Ceroid Lipofuscinosis molecular diagnosis. PLoS One. 2014;9(10):e109576. 25333361 10.1371/journal.pone.0109576 PMC4198115

[18] Brandenstein L , Schweizer M , Sedlacik J , Fiehler J , Storch S . Lysosomal dysfunction and impaired autophagy in a novel mouse model deficient for the lysosomal membrane protein Cln7. Hum Mol Genet. 2016;25(4):777‐791. 26681805 10.1093/hmg/ddv615

[19] Sharifi A , Kousi M , Sagné C , et al. Expression and lysosomal targeting of CLN7, a major facilitator superfamily transporter associated with variant late‐infantile neuronal ceroid lipofuscinosis. Hum Mol Genet. 2010;19(22):4497‐4514. 20826447 10.1093/hmg/ddq381 PMC3298853

[20] Mohammed A , O'Hare MB , Warley A , Tear G , Tuxworth RI . In vivo localization of the neuronal ceroid lipofuscinosis proteins, CLN3 and CLN7, at endogenous expression levels. Neurobiol Dis. 2017 Jul;103:123‐132. 28365214 10.1016/j.nbd.2017.03.015 PMC5441185

[21] Das AK , Becerra CH , Yi W , et al. Molecular genetics of palmitoyl‐protein thioesterase deficiency in the U.S. J Clin Invest. 1998;102(2):361‐370. 9664077 10.1172/JCI3112 PMC508894

[22] Das AK , Lu JY , Hofmann SL . Biochemical analysis of mutations in palmitoyl‐protein thioesterase causing infantile and late‐onset forms of neuronal ceroid lipofuscinosis. Hum Mol Genet. 2001;10(13):1431‐1439. 11440996 10.1093/hmg/10.13.1431

[23] Lyly A , Schantz von C , Salonen T , et al. Glycosylation, transport, and complex formation of palmitoyl protein thioesterase 1 (PPT1)—Distinct characteristics in neurons. BMC Cell Biol. 2007;8(1):22. 17565660 10.1186/1471-2121-8-22 PMC1906764

[24] Munroe PB , Greene ND , Leung KY , et al. Sharing of PPT mutations between distinct clinical forms of neuronal ceroid lipofuscinoses in patients from Scotland. J Med Genet. 1998;35(9):790. 10.1136/jmg.35.9.790 PMC1051443 9733046

[25] Lauronen L , Munroe PB , Järvelä I , et al. Delayed classic and protracted phenotypes of compound heterozygous juvenile neuronal ceroid lipofuscinosis. Neurology. 1999;52(2):360‐365. 9932957 10.1212/wnl.52.2.360

[26] Munroe PB , Mitchison HM , O'Rawe AM , et al. Spectrum of mutations in the batten disease gene, CLN3. Am J Hum Genet. 1997;61(2):310‐316. 9311735 10.1086/514846 PMC1715900

[27] Järvelä I , Autti T , Lamminranta S , Aberg L , Raininko R , Santavuori P . Clinical and magnetic resonance imaging findings in Batten disease: analysis of the major mutation (1.02‐kb deletion). Ann Neurol. 1997;42(5):799‐802. 9392580 10.1002/ana.410420517

[28] Zhong N , Wisniewski KE , Kaczmarski AL , et al. Molecular screening of Batten disease: identification of a missense mutation (E295K) in the CLN3 gene. Hum Genet. 1998;102(1):57‐62. 9490299 10.1007/s004390050654

[29] Haskell RE , Carr CJ , Pearce DA , Bennett MJ , Davidson BL . Batten disease: evaluation of CLN3 mutations on protein localization and function. Hum Mol Genet. 2000;9(5):735‐744. 10749980 10.1093/hmg/9.5.735

[30] Wang F , Wang H , Tuan H‐F , et al. Next generation sequencing‐based molecular diagnosis of retinitis pigmentosa: identification of a novel genotype‐phenotype correlation and clinical refinements. Hum Genet. 2014;133(3):331‐345. 24154662 10.1007/s00439-013-1381-5 PMC3945441

[31] Cortese A , Tucci A , Piccolo G , et al. Novel CLN3 mutation causing autophagic vacuolar myopathy. Neurology. 2014;82(23):2072‐2076. 24827497 10.1212/WNL.0000000000000490 PMC4118497

[32] Blumkin L , Kivity S , Lev D , et al. A compound heterozygous missense mutation and a large deletion in the KCTD7 gene presenting as an opsoclonus‐myoclonus ataxia‐like syndrome. J Neurol. 2012;259(12):2590‐2598. 22638565 10.1007/s00415-012-6545-z

[33] Staropoli JF , Karaa A , Lim ET , et al. A homozygous mutation in KCTD7 links neuronal ceroid lipofuscinosis to the ubiquitin‐proteasome system. Am J Hum Genet. 2012;91(1):202‐208. 22748208 10.1016/j.ajhg.2012.05.023 PMC3397260

[34] Kousi M , Anttila V , Schulz A , et al. Novel mutations consolidate KCTD7 as a progressive myoclonus epilepsy gene. J Med Genet. 2012;49(6):391‐399. 22693283 10.1136/jmedgenet-2012-100859 PMC3773914

