# Supplementary material for: Functional characterization of novel MFSD8 pathogenic variants anticipates neurological involvement in juvenile isolated maculopathy
Source: Clin Genet. 2019 Dec 12;97(3):426–36. doi: 10.1111/cge.13673 (PMC7064892; doi:10.1111/cge.13673)
Supplement: Supplementary file 1 — Supplementary Table S1 Primers used for MFSD8 variant confirmation and segregation analysis. Supplementary Table 2. Identified variants in IRD genes after filtering of WES results. Hez: heterozygous, AF: allele frequency (in %). *: not main transcript Supplementary Table 3. Primers used for MFSD8 cDNA sequencing. Supplementary Table 4. Primers used for MFSD8 expression analysis. Supplementary Table 5. Primers used to amplify the insert cloned into a minigene construct. Supplementary Table 6. Primers used to amplify cDNA for human mutant CLN7 Ile67Glufs*3 [file CGE-97-426-s001.docx]

**SUPPLEMENTARY FILES**

**Supplementary Table 1.** Primers used for *MFSD8* variant confirmation and segregation analysis.

| *MFSD8*_1_F | TTTAGTGCCACAGATGTGTCA |
| --- | --- |
| *MFSD8*_1_R | CAGGTTGTCTTTTGGTTGCCA |
| *MFSD8*_2_F | TCTTGGCCAAATGGTAGCTT |
| *MFSD8*_2_R | TTTAAAAGGGGATGAGACCAG |

**Supplementary Table 2**. Identified variants in IRD genes after filtering of WES results. Hez: heterozygous, AF: allele frequency (in %). *: not main transcript

| **Gene** | **Genomic coordinate (Hg38)** | **c.nomenclature** | **p.nomenclature** | **Effect** | **Transcript** | **Zygosity** | **AF gnomAD (ALL)** | **ClinVar** |
| --- | --- | --- | --- | --- | --- | --- | --- | --- |
| *MFSD8* | g.127939960AC>A | c.590del | p.Gly197ValfsTer2 | Frameshift | ENST00000296468 | HeZ | / | Not reported |
| *MFSD8* | g.127943749T>G | c.439+3A>C | p.Ile67Glufs*3 | Exon-skipping / Frameshift | ENST00000296468 | HeZ | / | Not reported |
| *ABCA4* | g.94043413G>A | c.3113C>T | p.Ala1038Val | Missense | ENST00000370225 | HeZ | 0.18% | Pathogenic |
| *CRB1* | g.197428984A>G | c.2480A>G | p.Asn827Ser | Missense* / Deep-intronic | ENST00000535699* | HeZ | 0.00078% | Not reported |
| *USH2A* | g.215844349A>G | c.9203T>C | p.Val3068Ala | Missense | ENST00000307340 | HeZ | 0.041% | Likely benign, uncertain signficance |
| *USH2A* | g.215934786T>C | c.7130A>G | p.Asn2377Ser | Missense | ENST00000307340 | HeZ | 0.38% | Benign, likely benign |
| *ZNF513* | g.27378305C>G | c.866G>C | p.Cys289Ser | Missense | ENST00000323703 | HeZ | 0.010% | Uncertain significance |
| *IFT172* | g.27454631C>A | c.3401G>T | p.Arg1134Leu | Missense | ENST00000260570 | HeZ | 0.54% | Benign, likely benign |
| *SNRNP200* | g.96297681T>C | c.1159A>G | p.Met387Val | Missense | ENST00000323853 | HeZ | 0.3% | Benign, uncertain significance |
| *AHI1* | g.135429942T>C | c.2432A>G | p.Asn811Ser | Missense | ENST00000265602 | HeZ | 0.0047% | Not reported |
| *CNGB1* | g.57901371T>A | c.2957A>T | p.Asn986Ile | Missense | ENST00000251102 | HeZ | 0.12% | Pathogenic, uncertain significance |
| *ABHD12* | g.25308475G>A | c.769C>T | p.Arg257Trp | Missense | ENST00000339157 | HeZ | 0.1% | Uncertain significance |

**Supplementary Table 3.** Primers used for *MFSD8* cDNA sequencing.

| *MFSD8*_CDNA_F | GCAGTGTAGGGTTTTCTGTAGTG |
| --- | --- |
| *MFSD8*_CDNA_R | GGGAAGTAGCACCAGCAGTA |

**Supplementary Table 4**. Primers used for *MFSD8* expression analysis.

| *MFSD8*_expr_F | CTACACTGCTGAGAAACATAGT |
| --- | --- |
| *MFSD8*_expr_R | TTTAGAGACTGAAGAGCATTATAAGA |
| *YWHAZ*_exp1F | GCAGAGAGCAAAGTCTTCTA |
| *YWHAZ*_exp1R | CTGATCGACAATCCCTTTCT |
| *GAPDH*_Exp1F | GTCGGAGTCAACGGATTT |
| *GAPDH*_Exp1R | GGCAACAATATCCACTTTACC |

**Supplementary Table 5**. Primers used to amplify the insert cloned into a minigene construct.

| *MFSD8*_MG_F | CAGCCTGGATGTCAGAGTGA |
| --- | --- |
| *MFSD8*_MG_R | AACCCAGGCAGGTAGAGGAT |

**Supplementary Table 6**. Primers used to amplify cDNA for human mutant CLN7 Ile67Glufs*3.

| *CLN7*_F | GCGAAGCTTGCCGGCCTGCGGAACGAAAGTGAA |
| --- | --- |
| *CLN7*_R | CGCGGATCCCTACATTTCCTTTTGGAGATATGGCCATATGGA |
